# The Design of Aluminum-Matrix Composites Reinforced with AlCoCrFeNi High-Entropy Alloy Nanoparticles by First-Principles Studies on the Properties of Interfaces

**DOI:** 10.3390/nano12132157

**Published:** 2022-06-23

**Authors:** Yu Liu, Guangping Zheng

**Affiliations:** 1 Research Institute of Light Alloy, Central South University, Changsha 410083, China; csuliuyu@csu.edu.cn; 2 Department of Mechanical Engineering, The Hong Kong Polytechnic University, Hung Hom, Kowloon, Hong Kong

**Keywords:** aluminum matrix composites, AlCoCrFeNi high-entropy alloy, interfacial behaviors, mechanical properties, first-principles calculations

## Abstract

The present work reports the interfacial behaviors and mechanical properties of AlCoCrFeNi high-entropy alloy (HEA) reinforced aluminum matrix composites (AMCs) based on first-principles calculations. It is found the stability of HEA-reinforced AMCs is strongly dependent on the local chemical compositions in the interfacial regions, i.e., those regions containing more Ni atoms (>25%) or fewer Al atoms (<20%) render more stable interfaces in the HEA-reinforced AMCs. It is calculated that the interfacial energy of Al(001)/Al_20_Co_19_Cr_19_Fe_19_Ni_19_(001) interfaces varies from −0.242 eV/Å^2^ to −0.192 eV/Å^2^, suggesting that the formation of interfaces at (100) atomic plane is energetically favorable. For those constituent alloy elements presented at the interfaces, Ni could stabilize the interface whereas Al tends to deteriorate the stability of interface. It is determined that although the HEA-reinforced AMCs have less yield strength compared to aluminum, their Young’s modulus is enhanced from 69 GPa for pure Al to 134 GPa. Meanwhile, the meaningful plasticity under tension could also be improved, which are related to the chemical compositions at the interfaces. The results presented in this work could facilitate the designs of compositions and interfacial behaviors of HEA-reinforced AMCs for structural applications.

## 1. Introduction

In the past decades, nanoparticle-reinforced aluminum matrix composites (AMCs) have attracted enormous attention because of their high specific strength, excellent wear resistance, as well as corrosion resistance [1,2,3,4]. Those AMCs have great potential for applications in aviation, transportation, manufacturing, and some other industries. Ceramic nanoparticles, such as Al_2_O_3_, TiB_2_, TiC, SiC, which possess high mechanical strength and stiffness were firstly proposed and introduced into aluminum to fabricate the AMCs [5,6,7,8], whose mechanical properties are closely related to the interfacial wettability and bonding behaviors of interfaces between the ceramic reinforcements and the Al matrix. In general, poor wettability may result in weak bonding at the interfaces, and the brittle nature of some ceramics would lead to a poor strengthening effect and a large loss in plasticity in the AMCs [9,10]. In this regard, intermetallic particles with thermal expansion coefficients close to those of Al, good ductility, and metallic nature were frequently utilized as reinforcements in AMCs [11,12,13]. Hence, the intermetallic particles such as Al_3_Ni, Al_3_Ti, as well as AlFe, have been widely studied to reinforce Al.

More recently, a new type of metallic material, high-entropy alloy (HEA), has been developed, which is composed of five or more principal alloy elements with equal or near equal-molar fractions [14,15,16,17,18]. Many of the reported HEAs possess a simple solid-solution structure, such as body-centered cubic (b.c.c) and face-centered cubic (f.c.c) crystal structures, and they possess high yield strength and hardness, and outstanding thermal stability [17,19,20]. Particularly, the HEAs can be fabricated into thermally-stable nanoparticles with diameters as small as 10 nm, which are ideal intermetallic nanoparticles for reinforcing aluminum alloys. Several HEAs have been incorporated into aluminum alloys to fabricate AMCs [21,22,23] through different synthesis routes, such as friction deposition (FD), spark plasma sintering (SPS), powder metallurgy (PM), and submerged friction stir processing (SFSP); Meanwhile, the mechanical properties were improved in these fabricated HEA-reinforced AMCs. Because of strong lattice distortion in their interiors and surfaces, the HEA nanoparticles can provide high volume fractions of surfaces that are more reactive and wettable to the aluminum matrix than other alloy reinforcements. 

It is well known that the interface between the reinforced particle and aluminum matrix plays an important role in determining the mechanical properties of AMCs, as the interface between the reinforcements and the matrix could transfer loads from the reinforcements to the matrix, or vice versa, and the interface may also become a potential source of cracks. In this regard, some experimental studies were carried out to understand the behaviors of interfaces between the HEA reinforcements and aluminum matrix. Yang et al. [24] studied the interfacial characteristics of HEA-reinforced Al-Mg matrix composites by underwater friction stir processing; Yuan et al. [25] studied the effect of heat treatment on the interfaces in the HEA particle reinforced AMCs. Those studies characterized the interface behaviors in micro- and nano-scales, and had provided some insights into the strengthening mechanisms of the HEA-reinforced AMCs. Nevertheless, those investigations performed only from experiments are very limited and have not fully revealed the roles of interfaces in the deformation of HEA-reinforced AMCs. To further tailor the mechanical properties of AMCs with HEA reinforcements, it is of great importance to understand the strengthening mechanisms in the atomic scales. To this end, the first-principles calculations, based on density functional theory (DFT), were frequently employed to elucidate the interfacial properties of metallic materials in atomistic scales; Particularly, the interfacial energy and mechanical strength were computed to predict and explain the interfacial stability and mechanical properties of multiphase materials. Recently, first-principles calculations have been successfully applied to study the interfaces of AMCs, such as TiB_2_/Al [26], TiC/Al [27], and Graphene/Al [28]. Therefore, it is desirable that the interfacial behaviors and mechanical properties of HEA-reinforced AMCs could be examed by the first-principles studies, providing guidelines for the design of HEA-reinforced AMCs with high performance.

Motivated by the needs of theoretical research on the properties of interfaces between the HEA reinforcements and aluminum matrix, in the present work, first-principles calculations were employed to investigate the interfacial behaviors and mechanical properties of the AMCs. The widely reported HEA particles of AlCoCrFeNi, which was determined to possess a b.c.c structure, was selected as the reinforcement embedded in the aluminum matrix in the formation of Al/AlCoCrFeNi interfaces. The interfacial stability, electronic properties, as well as mechanical properties of the AMCs are systematically studied.

## 2. Computational Methods

**Figure 1:**
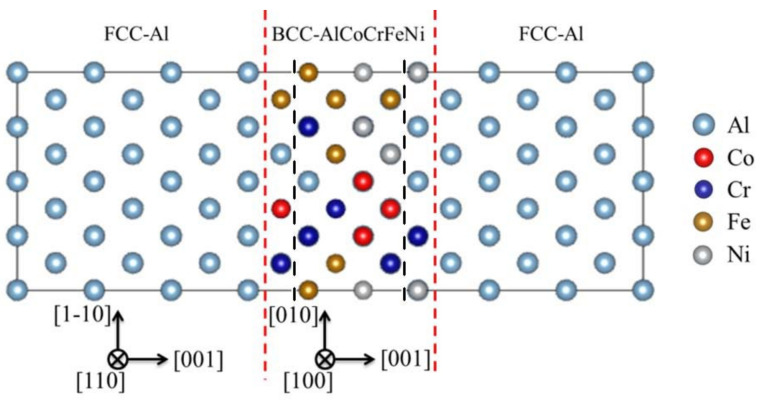
Typical configuration of supercell used for the calculations of HEA-reinforced AMCs; the red dash lines represent the interfaces between f.c.c-Al and the b.c.c-AlCoCrFeNi HEA. The interfacial regions are defined as those between the red and black dash lines.

In previous studies, the lattice parameters for the b.c.c AlCoCrFeNi HEA and f.c.c Al were determined to be 2.89 ± 0.05 Å [29] and 4.059 Å [30], respectively. In order to accommodate the lattice mismatch at the Al/AlCoCrFeNi interfaces, the (001)_fcc-Al_|(001)_bcc-HEA_ interface that aligned along [110]_fcc-Al_/[100]_bcc-HEA_ and [1–10]_fcc-Al_/[010]_bcc-HEA_, was built. Here, a configuration consisting of six b.c.c structured atomic layers of HEA (Al_20_Co_19_Cr_19_Fe_19_Ni_19_ with 96 atoms) and twelve f.c.c. structured aluminum layers (with 192 Al atoms) were used as the supercell for first-principles calculations, as shown in Figure 1. The HEA configurations were generated with an SQS method, which was compiled within the framework of ATAT software [31]. There were totally 26 configurations of supercells that had been utilized to simulate the chemical disorder in HEA.

**Figure 2:**
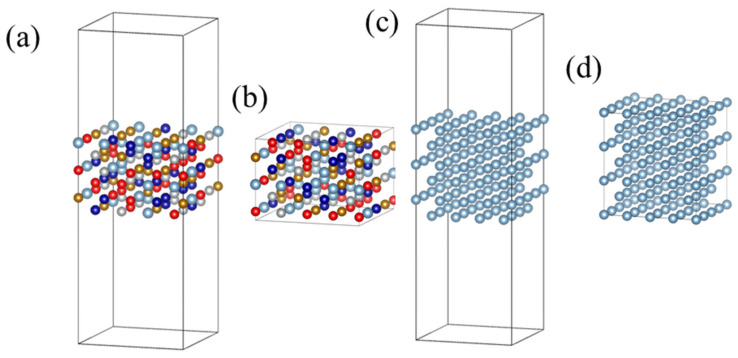
The supercells for model systems of (**a**) AlCoCrFeNi HEA with free surfaces, (**b**) bulk HEA, (**c**) Al with free surfaces, and (**d**) bulk Al, used in the calculation of surface energy and interfacial energy.

To evaluate the interfacial stability, the free energy of interface between the HEA layers and aluminum layers was also computed. The model of HEA with free surfaces was generated by removing the Al atomic layers in the supercell shown in Figure 1, and then, the HEA slab with (001) surfaces that consists of the remaining six HEA layers and the vacuum layers about 24 Å in thickness was obtained, as shown in Figure 2a. Further, the bulk model for HEA was obtained by removing the vacuum layers, as shown in Figure 2b. For the Al slab with (001) surfaces, six atomic layers stacked along [001] direction were constructed, and the vacuum layers with a thickness of about 26 Å were added, as shown in Figure 2c; and the bulk Al was constructed by removing the vacuum layers, as shown in Figure 2d.

The Vienna *ab-initio* simulation package (VASP) was employed to perform all the computations [32,33]. The generalized gradient approximation (GGA) method parameterized by Perdew, Burke, and Ernzerhof (PBE) was used for exchange-correlation functional [34]. Plane-wave energy cutoff of 350 eV was used in all the systems studied [35], and Monkhorst-Pack [36] k-point sampling scheme with the 3 × 3 × 1 grids was used for the calculations of HEA, Al and AMC model systems containing surfaces and interfaces, while the 3 × 3 × 4 k-point grids were used for the calculations of model systems of bulk HEA and Al. Meanwhile, a much denser k-point sampling scheme was used for the calculation of the density of states. The energy and force tolerance were set to be 1.0 × 10^−5^ eV and 1.0 × 10^−4^ eV·Å^−1^, respectively. To improve the calculation convergence and accuracy, the parameter of electron-state broadening of 0.2 eV was chosen, and Methfessel-Paxton method was used [37].

## 3. Results and Discussion

### 3.1. The Stability of HEA-Reinforced AMCs

**Figure 3:**
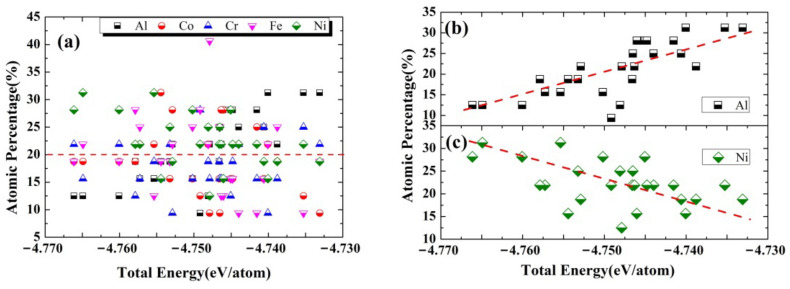
The total energy of HEA−reinforced AMCs against the chemical compositions of (**a**) all constituent alloy elements, (**b**) Al, and (**c**) Ni at the Al/AlCoCrFeNi interfaces.

Based on the generated 26 configurations for the supercells simulating the HEA-reinforced AMCs, the total energy of the system can be obtained after it is fully relaxed. The total energy is found to vary from −4.77 eV/atom to −4.73 eV/atom, which is closely related to the chemical composition at the Al/AlCoCrFeNi interfaces, as shown in Figure 3. It is found that with increasing Al concentration or decreasing Ni concentration near the interface, the total energy of the system increases, whereas the concentrations of Fe, Co, and Cr do not much affect the total energy of the HEA-reinforced AMCs.

Figure 3 indicates that the Al/AlCoCrFeNi interfaces become more stable or the interaction between the atomic surface layers of HEA and the adjacent Al layer could be stronger, when the Al/AlCoCrFeNi interfaces possess more Ni or less Al atoms. As reported, the electronegativity of Cr, Co, Ni, Fe, and Al atoms are 1.66, 1.88, 1.91, 1.83, and 1.61 [38], respectively, meaning that Ni atoms possess a better ability to attract electrons from the surrounding Al atoms than that of other metal atoms, i.e., the charge transfer between Ni and Al atoms is the strongest, which tends to reduce the total energy of the HEA-reinforced AMCs. Thus, the system with a higher Ni concentration (>20%) in the interfacial regions (the regions between the red and black dash lines in the model system shown in Figure 1 could be more stable in equilibrium, as shown in Figure 3c. Meanwhile, since Al atoms in the matrix and HEA are with the f.c.c and b.c.c crystal structures at their equilibrium states, respectively, the presence of Al atoms at the interfaces will reduce the stability of the HEA-reinforced AMCs. Therefore, when more Al atoms appear at the interface, the HEA-reinforced AMCs could be less stable and possesses high total energy, as shown in Figure 3b.

### 3.2. Electronic Properties

#### 3.2.1. Differential Charge Density

As mentioned above, the stability of the interface between metals or alloys is strongly related to the electronic structures around the interface. The charge distribution at the interface is one of the most important characteristics of electronic structures that could affect interfacial stability. The differential charge density, Δρ, is calculated for further analysis on the stability of Al/AlCoCrFeNi interfaces, which can be defined by the equation below,

(1) $${\Delta \rho =\rho }_{\mathrm{total}}\;-{\;\rho }_{\mathrm{Al}\;}\;-{\;\rho }_{\mathrm{Co}}\;-{\;\rho }_{\mathrm{Cr}}-{\;\rho }_{\mathrm{Fe}\;}-{\;\rho }_{\mathrm{Ni}}$$

where ρ_total_ is the total charge density of the AMC model system, ρ_Al_, ρ_Co_, ρ_Cr,_ ρ_Fe,_ and ρ_Ni_ are the charge densities of isolated Al, Co, Cr, Fe, and Ni, respectively.

**Figure 4:**
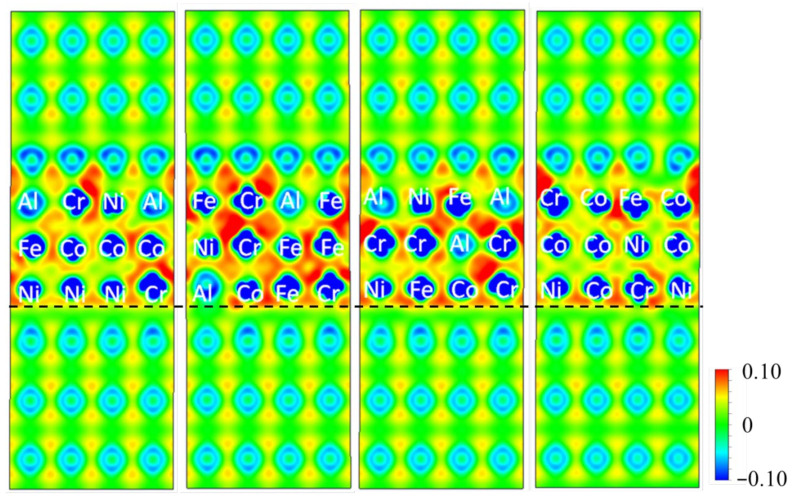
Four slices of isosurface plots of differential charge density Δρ within the plane perpendicular to the (001) Al layers.

Figure 4 presents four slices of isosurface plots of the differential charge density around the Al/AlCoCrFeNi interface within the cross-sectional area perpendicular to the (001) atomic plane of f.c.c-Al layers, where the atoms in the distorted HEA layers are also indicated. In the plots, the interface between the Al layers and the HEA layers is marked with a black dashed line, as shown in Figure 4. A weak bonding between Al atoms in the HEA layer and Al atoms in the Al layer can be identified since the differential charge density persistent to those Al atoms is close to zero, which is consistent with the above-discussed results that the interfaces with more Al atoms possess high free energy and are unstable. Moreover, it can be also observed that the bonding behaviors between Co, Cr, Fe, or Ni in the HEA layer and the Al atoms in the Al layer present limited difference. It probably results from the sliced planes that are perpendicular to the (001) atomic plane of f.c.c-Al layers, and the centers of those in the severely distorted b.c.c-HEA layers are not shown in the isosurface plots. Therefore, the densities of states (DOS) related to the interface have to be calculated to evaluate the bonding of Al and HEA layers.

#### 3.2.2. Density of States

**Figure 5:**
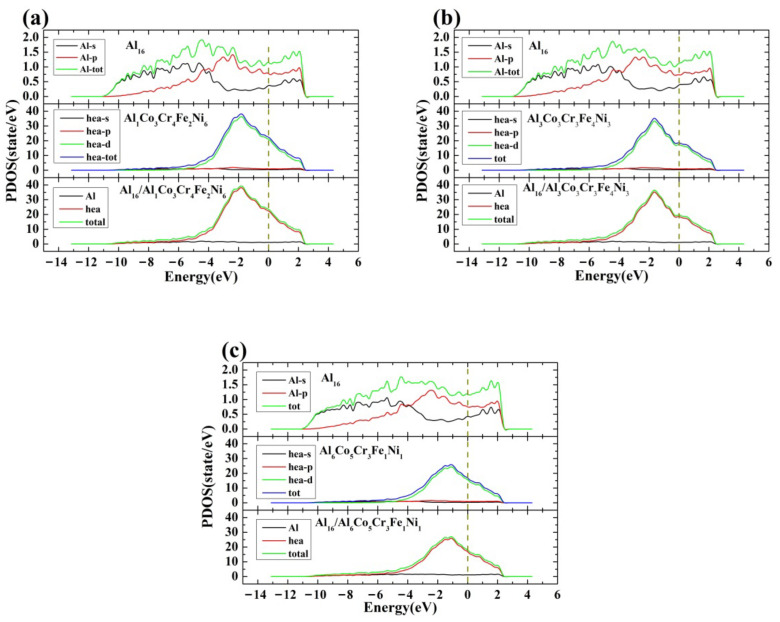
The computed layer-projected densities of states for the interfacial regions with three different Al (or Ni) concentrations, (**a**) Al_16_/Al_1_Co_3_Cr_4_Fe_2_Ni_6_, (**b**) Al_16_/Al_3_Co_3_Cr_3_Fe_4_Ni_3_, (**c**) Al_16_/Al_6_Co_5_Cr_3_Fe_1_Ni_1_; the dashed line refers to the Fermi level.

The layer-projected densities of states (LPDOS) that represent the electronic states of different orbitals of atoms in the interfacial regions are further utilized to study the mechanisms of bonding close to the interface between the Al and HEA layers. As revealed in Section 3.1, the concentrations of Al and Ni in the HEA layers play a key role in determining the stability of interface. The LPDOS for the interfacial regions with three different Al (or Ni) concentration gradients are computed, and the results are shown in Figure 5a–c, respectively. It is obvious that the chemical disorder strongly affects the density of states; Also, it can be found the total LPDOS is mainly attributed to the HEA layer, which mostly results from the d-orbitals for the HEA layer. Besides, obvious hybridizations at the energy level of −10~3 eV between p- and d-orbitals for HEA layers and s- and p-orbitals for Al layers are observed. Based on the magnitude of PDOS, one can deduce that the hybridization strength is enhanced with increasing Ni content or decreasing Al content at the interfaces. Moreover, with increasing Ni content or decreasing Al content at the interface, the total PDOS slightly shifts to lower energy levels, which could be mainly attributed to the existence of HEA layers. Therefore, the electronic structure discussed above further demonstrates that there are enhanced interactions between the HEA and Al layers close to the interface where the segregation of Ni or the depletion of Al could lead to better stability of interface.

### 3.3. The Interfacial Energy

To understand the stability of HEA-reinforced AMCs, it is of great importance to determine the interfacial energy for the formation of interfaces between HEA and aluminum layers. In general, the interfacial energy $\gamma_{\mathrm{int}}$ is computed by using the equation below [39],

(2) $$\gamma_{int}=\frac{E_{Al/HEA}-E_{Al}^{bulk}-E_{HEA}^{bulk}}{2A}{\;-\;\gamma }_{Al}\;{\;-\;\gamma }_{HEA\;}$$

where *A* is the area of the interface, *E_Al/HEA_* is the total energy of HEA-reinforced AMCs, $E_{Al}^{bulk}\;$ and $\;E_{HEA}^{bulk}\;$ are the total energies of bulk *Al* and bulk HEA, ${\;\gamma }_{Al}\;$ and ${\;\gamma }_{HEA}\;$ are surface energies of *Al* and HEA layers with free surfaces, respectively. To figure out the interfacial energy, the surface energy ${\;\gamma }_{sur}\;,\;$ i.e., either ${\;\gamma }_{Al}\;$ or ${\;\gamma }_{HEA}\;,\;$ is calculated by using the equation below [40],

(3) $$\gamma_{sur}=\frac{E_{sur}{\;-\;E}_{bulk}}{2A}$$

where *E_sur_* is the total energy of the Al or HEA layers with free surfaces, and *E_bulk_* is either $E_{Al}^{bulk}\;$ or $\;E_{HEA}^{bulk}$. The supercells of model systems for the calculations of Equations (2) and (3) are presented in Figure 3. Similarly, a total of 26 configurations are used for the HEA layers in the model systems.

**Figure 6:**
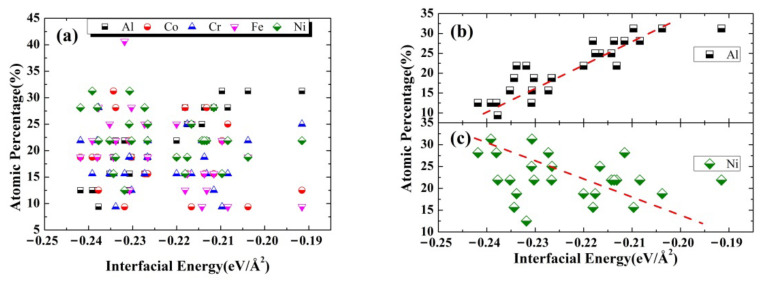
The calculated interfacial energy of Al (001)/AlCoCrFeNi (001) against chemical compositions of (**a**) all constituent alloy elements, (**b**) Al, and (**c**) Ni in the interfacial regions.

Firstly, the surface energy of Al (001) is computed to be ${\;\gamma }_{Al}=$0.058 eV/Å^2^ (~0.928 J/m^2^), which is well consistent with that (0.9 J/m^2^) obtained in previous work [41]; Whereas the calculated surface energy of Al_20_Co_19_Cr_19_Fe_19_Ni_19_ (001) is determined to be 2–3 times higher than that of Al (001), which ranges from ${\;\gamma }_{HEA}=$0.126 eV/Å^2^ to ${\;\gamma }_{HEA}=$0.161 eV/Å^2^. Then, based on the obtained surface energies, the interfacial energy ${\;\gamma }_{int}$ is computed to range from −0.242 eV/Å^2^ to −0.192 eV/Å^2^. The negative interfacial energy could indicate that the formation of the Al(001)/Al_20_Co_19_Cr_19_Fe_19_Ni_19_(001) interface is energetically favorable. Figure 6 shows the calculated interfacial energy against chemical compositions in the interfacial regions, demonstrating the strong dependence of interfacial energy on the local chemical compositions. The interfacial regions that contain more Ni or less Al atoms lead to lower interfacial energy, suggesting that Ni and Al atoms at the interfaces are prone to increasing and decreasing the stability of interfaces, respectively. The results agree well with those discussed in Section 3.1 and Section 3.2.

### 3.4. Mechanical Properties

**Figure 7:**
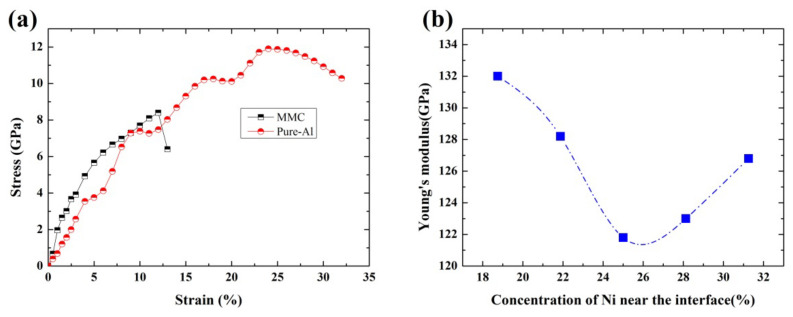
(**a**) The computed strain-stress curves for AlCoCrFeNi HEA reinforced AMCs and pure aluminum, (**b**) Young’s modulus of AMCs against the Ni concentration in the interfacial regions.

The mechanical properties of HEA-reinforced AMCs are determined from the strain-stress relations of the model system under tensions along [001] direction, which is the most stable model system (the one with the lowest free energy) selected from those with various HEA configurations. For the purpose of comparison of the mechanical properties, those of pure aluminum are also determined from the strain-stress relations of the pure-Al model system under tensions along [001] direction. The obtained strain-stress curves for the two model systems are presented in Figure 7a.

The Young’s modulus is calculated based on the strain-stress relations in the elastic region, as shown in the tensile curves in Figure 7a, which are estimated to be 134 GPa and 69 GPa for HEA-reinforced AMCs and pure Al, respectively. Further, the yield strength is determined from the crossing point between the tensile curve and a straight line which has a slope equal to Young’s modulus and is at an offset of 0.2% strain from the origin. It is found that the yield strength is about 2.7 GPa and 3.73 GPa for the HEA-reinforced AMC and pure Al, respectively. An elongation of 12% or 10% can be determined for the HEA-reinforced AMC and pure Al, respectively, which is the upper limit of tensile strain in the strain-hardening regions at the tensile curve and could be a measure of the meaningful plasticity (ductility) of the system. Based on the mechanical properties determined above, it is suggested that the introduction of HEA reinforcements into the Al matrix would lead to a loss of yield strength while the Young’s modulus and plasticity could be improved, as compared with those of pure aluminum.

The mechanical properties dependence on interfacial behaviors in HEA-reinforced AMCs is further analyzed, which could be closely related to the chemical compositions in the interfacial regions. Figure 7b presents Young’s modulus against the concentration of Ni atoms in the interfacial regions. It is found that the enrichment or depletion of Ni could be favorable for the enhancement of Young’s modulus of the AMCs; whereas when the concentration of Ni in the interfacial regions is about 25%, the enhancement in the Young’s modulus is the lowest. 

**Figure 8:**
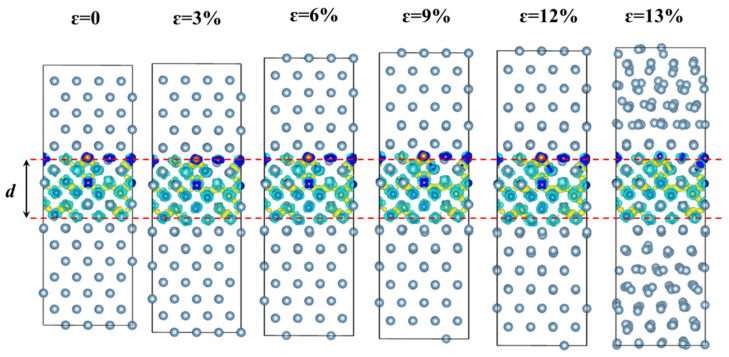
The evolution of atomic structure of HEA-reinforced AMC under various tensile strains applied along [001] direction.

To further understand the fracture behavior of the HEA-reinforced AMCs, the atomic structures under different applied strains are shown in Figure 8, together with the differential charge density as calculated in Section 3.2.1. As shown in Figure 8, the thickness, *d*, of the HEA layers varies little under the applied strains, meaning that the Al layers mainly withstand the strain applied on the HEA-reinforced AMCs. It could be caused by the fact that the bonding strength among the HEA layers is much higher than that of Al layers. Interestingly, when the fracture occurs in HEA-reinforced AMCs under an applied strain of 13%, atoms in the Al layers are strongly distorted due to the dislocation multiplications, while those Al atoms close to the interface distort little. Such interfacial behavior further indicates the bonding strength between the HEA layer and the Al layer is also stronger than that among the Al layers, and the formation of AlCoCrFeNi HEA reinforced AMCs is highly feasible.

### 3.5. Discussion and Remarks on the Designs of HEA-Reinforced AMCs

The interfacial behaviors and mechanical properties of the HEA-reinforced AMCs are found to strongly depend on the local chemical compositions in the interfacial regions. Several remarks are derived, as follows: First, since the AlCoCrFeNi HEA studied in this work has an equal molar ratio of constituent alloy elements, it is an effective strategy that the contents of Al and Ni atoms of the HEA could be modified to further stabilize the interfaces, thereby tuning the HEA-reinforced AMCs to have good mechanical properties. For a better application of AlCoCrFeNi HEA in reinforcing AMCs, it is of great importance to develop new AlCoCrFeNi HEA with higher Ni content (>25%) or lower Al content (<20%) while keeping its b.c.c crystal structure. Second, based on the computed negative interfacial energy of Al(001)/Al_20_Co_19_Cr_19_Fe_19_Ni_19_(001) interface, several experimental results can be explained. Li et al. [42] fabricated an Al-matrix composite reinforced by Al_0.8_CoCrFeNi HEA particles via multi-pass friction stir processing (FSP). The average grain size of the FSPed composites decreased from 4.6 μm of Al matrix to 2.8 μm, which was mainly attributed to the HEA particle stimulated nucleation mechanism. Also, Yang et al. [24] prepared the 5083 Al matrix composites reinforced by 10 vol% of AlCoCrFeNi HEA particles by submerged friction stir processing (SFSP). The Al matric was found to have equiaxed fine grains with a mean size of 1.2 μm, which was ascribed to HEA particle induced stimulated nucleation. Thus, it is suggested that the AlCoCrFeNi HEA reinforcements could be used as grain refiners during the solidification processes or as the recrystallization nucleus during the hot working processes of Al-based materials.

Third, since the HEA-reinforced AMCs possess a low yield strength and a significantly improved Young’s modulus in comparison with the pure aluminum, the AlCoCrFeNi HEA reinforced AMCs could be utilized in a high-modulus application scenario.

## 4. Conclusions

In this work, the interfacial behaviors and mechanical properties of the AlCoCrFeNi HEA-reinforced AMCs are investigated by first-principles calculations. It is found that the stability of Al/Al_20_Co_19_Cr_19_Fe_19_Ni_19_ interfaces would be enhanced when the interfacial regions contain more Ni atoms or fewer Al atoms. The formation of the interface is energetically favorable since the computed interfacial energy varies from −0.242 eV/Å^2^ to −0.192 eV/Å^2^. The introduction of HEA reinforcements would cause a loss of yield strength while leads to a much enhanced Young’s modulus and good plasticity of AMCs as compared to those of pure aluminum. Based on the aforementioned results, the HEA-reinforced AMCs with enhanced mechanical properties could be developed for structural applications.

## Data Availability

The data presented in this study are available on request from the corresponding author.
